# Intramesenteric Steroid Treatment for Steroid- Refractory Gastrointestinal Graft Versus Host Disease

**DOI:** 10.5505/tjh.2012.57855

**Published:** 2012-12-05

**Authors:** Aynur Uğur Bilgin, Pervin Topcuoğlu, Tanzer Sancak, Nahide Konuk, Mutlu Arat

**Affiliations:** 1 Necmettin Erbakan University, Meram Faculty of Medicine, Division of Hematology, Department of Internal Medicine, Konya, Turkey; 2 Ankara University, School of Medicine, Division of Hematology, Department of Internal Medicine, Ankara, Turkey; 3 Ankara University, School of Medicine, Division of Radiology, Ankara, Turkey

**Keywords:** Steroid-refractory GVHD, Intramesenteric steroid

## Abstract

Currently, steroid-refractory severe gastrointestinal (GI) graft versus host disease (GVHD) is among the most important complications of allogeneic transplantation, and as yet there is no standard approach to its treatment. Herein we report two cases with steroid-refractory GI GVHD that received intramesenteric steroid treatment. In both cases the frequency and volume of diarrhea resolved completely following intramesenteric methylprednisolone (MP) injection. In conclusion, intra-arterial steroid injection might be an alternative treatment approach for steroid-refractory GI GVHD.

**Conflict of interest:**None declared.

## Abstract (tr)

Günümüzde, steroid dirençli gastrointestinal (Gİ) graft versus host hastalığı (GvHH) allogeneik transplantasyon yapılan hastalarda izlenen en sık komplikasyonlardan biridir ve henüz standart bir tedavi yaklaşımı yoktur. Burada, steroid refrakter GvHH olan iki vakanın intramezenterik steroid uygulamasına dair sonuçları bildirilmiştir. Her iki hastada intramezenterik metilprednizolon (MP) enjeksiyonunu takiben diarenin sıklığı ve miktarı tamamen düzelmiştir. Sonuç olarak, intra-arteriel steroid verilimi bu tip olgularda alternative bir tedavi yaklaşımı olabilir.

## INTRODUCTION

Steroid-refractory severe gastrointestinal (GI) graft versus host disease (GVHD) is one of the most challenging complications of allogeneic hematopoietic cell transplantation (allo-HCT), and is associated with very high morbidity and mortality rates. Treatment with various salvage regimens has been used, but the results have not been satisfactory. In recent years, regional administration of steroids directly through the arteries that supply blood to the liver and intestines has resulted in varying degrees of effectiveness [1,2,3]. Herein we present 2 cases with steroidrefractory severe GI GVHD in which significantly stable recovery followed a single intra-arterial injection of methylprednisolone (MP) through the superior and inferior mesenteric arteries.

## CASE 1

A 44-year-old male patient with AML underwent allo-HCT from an HLA-identical sister following a myeloabla tive conditioning regimen. Symptoms of grade III (skin,liver, and GI) acute GVHD emerged on d 30 of hematologicalrecovery, which were confirmed based on pathologicalexamination. Skin symptoms resolved following IV MP 2mg·kg^–^1·d^–^1, but frequent diarrhea (maximum volume: 6L d^–^1) persisted. Due to exacerbation of GI symptoms, i.e.severe abdominal pain and hematochezia, antithymocyteglobulin was initiated on d 35 of hematological recovery.As the patient’s diarrhea did not abate and he had an elevatedbilirubin level and metabolic disorders, 4 weeks ofinfliximab 10 mg kg^–^1 QWK was started on d 41 of hematological recovery.

**Figure 1:**
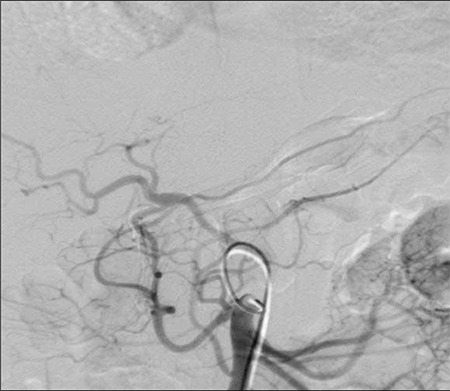
Digital subtraction angiography of the hepatic artery.

**Figure 2:**
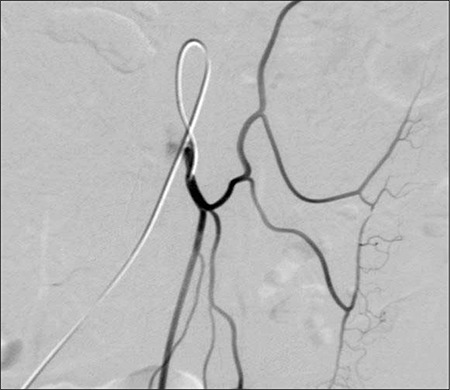
Digital subtraction angiography of the superior mesenteric artery.

Despite of all the immunosuppressive treatments, thepatient’s diarrhea continued at the rate of 3 L d^–^1; therefore,intramesenteric arterial MP infusion was administeredon d 54 of hematological recovery. Briefly, underdigital subtraction angiography (DSA) guidance (MultistarPlus/T.O.P., Siemens AG, Forchheim, Germany), via theright femoral artery No. 5 French catheters (Contra 2, ImagerII, Boston Scientific/Medi-Tech, Annacotty, Limerick,Ireland) were placed with their mesenteric tips. Selectiveviews were obtained via injection of 6-8 mL of non-ioniccontrast agent (Iomeron 400 mg/dl, Bracco SpA, Milan, Italy) in order to precisely visualize the vascular supply ofthe superior and inferior mesenteric arteries. Additionally,selective celiac angiography was performed in order to determineif there was anatomical variation. Selectively, 1 mgkg^–^1 of MP in 10 mL of sterile saline was infused over the course of 3 min into the superior and inferior mesenteric arteries (Figures 1 and 2). 

**Figure 3:**
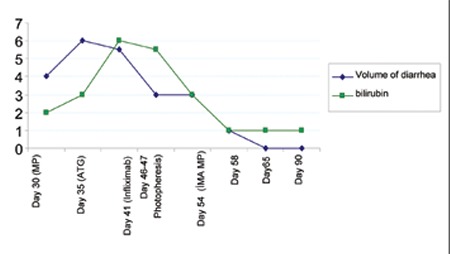
Volume of diarrhea and the bilirubin level prior to and after regional steroid injection in case 1.

The patient’s diarrhea decreased in volume and frequency(1 L d^–^1 2 d) after administration of intra-arterialsteroid. The patient’s metabolic and clinical status beganto recover on the fourth day. The symptoms of GI GVHDwere completely resolved 90 d post treatment (Figure 3)and he was discharged with CsA alone as immunosuppressive therapy. Unfortunately, the patient died due tosystemic infection 224 d post treatment.

## CASE 2

A 56-year-old male with idiopathic myelofibrosis underwent allo-HCT from an HLA identical sibling donor and non-myeloablative conditioning in November 2005. Donor lymphocyte infusion was administered on 10 May 2006 because of increasing-donor type chimerism. The patient developed grade II acute GVHD in the skin and liver 1 month after donor lymphocyte infusion (DLI). Steroid plus CsA was administered, but acute GVHD progressed to extensive chronic GVHD, involving the eyes, the GI system, and bone marrow. The results of skin and colon biopsies confirmed grade III skin and GI GVHD. 

Oral budesonide, mycophenolate mofetil, sirolimus, and extracorporeal photopheresis (ECP) were added for the treatment of GVHD. Despite all treatment, GI GVHD could not be controlled. We administered intramesenteric steroid infusion, as described in case 1. Marked improvement in the patient’s symptoms and a decrease in the volume of diarrhea was noted 72 h following the procedure. Complete resolution of diarrhea occurred 15 d after steroid infusion. As of 3 years following the procedure (at the time this manuscript was prepared) the patient did not have recurrence of GI GVHD.

## DISCUSSION

Corticosteroids are currently used as front-line therapy for acute and chronic GVHD; however, less than 50% of patients, especially those with grade II-IV acute GVHD, achieve durable response following initial treatment. Mortality is very high in patients with steroid-refractory disease and therapeutic options are limited. Several immunosuppressive drugs, including mycophenolate mofetil, sirolimus, pentostatin, rituximab, and ECP, have been used as salvage regimens [4]. To date, however, the most effective strategy remains unknown. 

GI GVHD has similar features as severe ulcerative colitis (UC). Steroid administration via the superior and inferior mesenteric arteries is very effective in UC patients that are unresponsive to conventional therapy [5,6]. An extensive review of literature showed that there are several publications on the use of regional steroids in GVHD. Sato et al. [1] were the first to report the beneficial role of this approach and subsequently many other case studies reported that similar patients were also successfully treated with the same approach [2,3,7]. Encouraged by these reports we used the same approach to treat steroid-refractory severe GI GVHD and observed complete resolution of the symptoms of GI GVHD within a few days of administration of the treatment. Unlike previously reported patients, the 2 presented cases were not only refractory to high-dose systemic steroid treatment, but were also refractory to secondary salvage therapy. 

In the patients with refractory to systemic steroids, the mechanisms underlying regional and low-dose intrames-enteric steroid infusion are not exactly known. Rogler and Schottelius reported a decrease in the number and affinity of steroid receptors in GI tract mucosa in patients with inflammatory bowel disease [8,9]. Administration of regional steroid infusion versus systemic may cause higher concentration of steroids in the mesenteric circulation and inflammatory mucosa in patients with GI GVHD. Hence, regional steroid application may overcome the refractoriness caused by a decrease in the number and affinity of steroid receptors. 

In conclusion, the two presented cases with severe GI GVHD, and refractoriness to steroid and secondary salvage therapies responded dramatically to regional administration of steroids via the mesenteric artery. A single dose of intramesenteric artery steroid infusion appears to be effective, even in patients that have not responded to multiple treatment regimens, and is not associated with extensive systemic immunosuppression. Nonetheless, we cannot exclude that the treatment responses observed in the two presented cases were not due to the delayed therapeutic effect of their initial steroid treatment or other immunosuppressive agents. However, the resolution of GI symptoms within 72 h of intra-arterial administration demonstrate the efficiency of this novel treatment. Based on the present findings, intra-arterial administration of steroids is easy, reliable, and safe, and can be considered a first-line treatment, especially for avoiding severe systemic immunosuppression in patients with severe GI GVHD. On the other hand, controlled studies are required to exclude the role of previous immunosuppressive treatments. 

Informed consent was obtained. 

**Conflict of Interest Statement**

The authors of this paper have no conflicts of interest, including specific financial interests, relationships, and/ or affiliations relevant to the subject matter or materials included.
